# Dapagliflozin Ameliorates Diabetic Kidney Disease *via* Upregulating Crry and Alleviating Complement Over-activation in *db/db* Mice

**DOI:** 10.3389/fphar.2021.729334

**Published:** 2021-10-12

**Authors:** Dong-Yuan Chang, Xiao-Qian Li, Min Chen, Ming-Hui Zhao

**Affiliations:** ^1^ Renal Division, Department of Medicine, Peking University First Hospital, Beijing, China; ^2^ Key Laboratory of Renal Disease, Ministry of Health of China, Beijing, China; ^3^ Research Units of Diagnosis and Treatment of Immune-Mediated Kidney Diseases, Chinese Academy of Medical Sciences, Beijing, China

**Keywords:** sodium glucose co-transport-2 (SGLT2) inhibitors, dapagliflozin, diabetic kidney disease, complement over-activation, complement receptor type 1-related protein y (Crry), hypoxia inducible factor-1α (HIF-1 α)

## Abstract

Sodium-glucose cotransporter 2(SGLT2) inhibitors show prominent renal protective effect in diabetic kidney disease (DKD), anti-inflammatory effect being one of its key mechanisms. Over-activation of the complement system, a crucial part of innate immunity, plays an important role in DKD. We aimed to investigate the effect of SGLT2 inhibitors on alleviating complement over-activation in DKD. *Db/db* mice were randomly divided into two groups, with 7 mice in each group treated with dapagliflozin and vehicle respectively, and 7 mice in *m/m* mice group. Laboratory and renal pathological parameters were evaluated. Mouse proximal tubular epithelial cells (MPTECs) were cultured and treated with high glucose. Dapagliflozin and dimethyloxallyl glycine (DMOG) were added as conditional treatment. Dapagliflozin-treated *db/db* mice showed significantly lower urinary albumin than vehicle-treated ones. Besides typical glomerular and tubulointerstitial injury, both C3b and membrane attack complex (MAC) depositions were significantly attenuated in dapagliflozin-treated *db/db* mice. The expression of complement receptor type 1-related protein y (Crry), a key complement regulator which inhibits complement over-activation, was significantly upregulated by dapagliflozin. Dapagliflozin-mediated Crry upregulation was associated with inhibition of HIF-1α accumulation under high glucose. When HIF-1α expression was stabilized by DMOG, the protective effect of dapagliflozin *via* upregulating Crry was blocked. In conclusion, dapagliflozin could attenuate complement over-activation in diabetic mice *via* upregulating Crry, which is associated with the suppression of HIF-1α accumulation in MPTECs.

## Introduction

With the increasing prevalence of diabetes, diabetic kidney disease (DKD) has surpassed glomerulonephritis and become the leading cause of chronic kidney disease and end-stage renal disease (ESRD) (Zhang et al., 2016; Wang et al., 2017; Ma, 2018). However, the effective prevention and treatment of DKD were still limited.

The sodium-glucose cotransporter 2 (SGLT2) inhibitor is a novel anti-diabetic medication, targeting renal proximal tubules to reduce glucose reabsorption, resulting in increased urinary glucose excretion and anti-hyperglycemic effects (Uthman et al., 2018; Wilcox et al., 2018). A number of recent clinical trials demonstrated prominent renal protective effects of SGLT2 inhibitors (Wanner et al., 2016; Neal et al., 2017; Wiviott et al., 2019). SGLT2 inhibitors have already been recommended as the first-line medication for patients with chronic kidney diseases for the management of type 2 diabetes (Kidney Disease: Improving Global Outcomes Diabetes Work, 2020). However, the renal protective mechanism of SGLT2 inhibitors has not been fully clarified. Cumulating evidence suggests that, besides the anti-hyperglycemic effect, SGLT2 inhibitors have a remarkable anti-inflammatory role in DKD (Ashrafi Jigheh et al., 2019; Hasan et al., 2020; Pirklbauer et al., 2020).

Immune inflammation, especially innate immunity, plays an important role in the pathogenesis of DKD (Tesch, 2017; Tang and Yiu, 2020). The complement system is an important part of innate immunity, with functions of cleaving pathogens, clearing immune complexes and participating in inflammatory response (Kolev et al., 2014). The complement system can be activated through three pathways including classical, lectin and alternative pathway. Complement regulatory proteins, mainly including human membrane cofactor protein (MCP, CD46), decay accelerating factor (DAF, CD55) and CD59, inhibit the key steps of complement cascade activation, thus protect tissue cells from damage by complement over-activation. Complement receptor type 1-related protein y (Crry) is a key complement regulatory protein of mice with similar function to CD46 and CD55 in human (Nangaku, 1998; Thurman et al., 2006; Miwa et al., 2007). There is growing evidence that over-activation of the complement system plays an important role in the development of DKD (Li et al., 2019a; Li et al., 2019b; Huang et al., 2019; Sun et al., 2019), including two aspects of mechanism. One is that advanced glycation of proteins generates neo-epitopes to which lectin pathway pattern recognition molecules bind. Meanwhile, glycation-induced dysfunction or inactivation of complement regulatory proteins is another important mechanism for the complement over-activation in DKD (Qin et al., 2004; Ostergaard et al., 2007).

On the other hand, in early and advanced stages of DKD, hypoxia in renal proximal tubules is a common feature (Friederich-Persson et al., 2013; Liu et al., 2017). Hypoxia-inducible factor (HIF)-1α is a key molecule playing an adaptive reaction in hypoxic conditions. The diabetic kidney is characterized by abnormalities in HIF-1α signaling. Accumulated HIF-1α was associated with renal tissue damage including inflammation and fibrosis (Baumann et al., 2016; Nayak et al., 2016; McGettrick and O'Neill, 2020). Recent studies suggested HIF-1α could be a therapeutic target of SGLT2 inhibitors in DKD, and multiple molecular mechanisms were investigated but still unclear (Bessho et al., 2019; Cai et al., 2020; Li et al., 2020). Pandya et al. (2016) found that there was potential association between HIF-1α and CD55 in respiratory epithelium. Therefore, whether there is similar regulatory relationship in kidneys is of interest.

In the present study, we hypothesized that the SGLT2 inhibitor could alleviate complement over-activation in DKD *via* upregulating Crry, which was associated with the suppression of HIF-1α accumulation.

## Materials and Methods

### Animal Experimentation

Male *db/db* (Lepr^*db/db*^) and *m/m* mice (C57BL/KsJ mice) were obtained from Nanjing Biomedical Research Institute of Nanjing University. The mice were housed in the Animal Center of Peking University First Hospital with a 12-h light/dark cycle allowing free access to food and water when they were 3 weeks old. After 1 week (at the age of 4 weeks), *db/db* mice were randomly divided into two groups, with 7 mice for each group. For dapagliflozin-treated group, the dosage of dapagliflozin (AstraZeneca Pharmaceuticals LP) was 1 mg/kg/day and the solvent was 0.5% methylcellulose. The control groups received solvent as vehicle without active pharmacological effect.

24-h urine was collected to measure urinary albumin at the 8th and 14th week. Mice were placed in a 24-h metabolic cage for urine collection. Urinary albumin levels were measured by a mouse albumin ELISA kit (Bethyl Laboratories, Montgomery, TX, United States). Urinary creatinine levels were assayed by a Creatinine Assay Kit (DICT-500; BioAssay Systems, CA, United States). The mice were sacrificed at the age of 14 weeks. No adverse effect was found in dapagliflozin-treated *db/db* mice. All animal experiments were approved by the Laboratory Animal Ethics Committee of Peking University First Hospital (No. 202039).

### Morphological Analysis and Immunohistochemistry

Formalin-fixed paraffin-embedded kidney tissue sections were stained with periodic acid-Schiff (PAS, BA-4080B; BASO, Zhuhai, China) solution. The glomerular injury was evaluated by mesangial matrix fraction. The percentage of mesangial matrix expansion area to the glomerular tuft area was calculated in at least 20 glomeruli per renal section randomly selected and digitized under ×400 magnification (Yiu et al., 2016). Image-Pro Plus software V.6.0 (Media Cybernetics, Bethesda, MD) was used to analyze. Tubulointerstitial injury was scored from 0 to 5 according to the percentage of damaged tubulointerstitium (tubular dilation, vacuolar degeneration, interstitial inflammation and cast formation): 0, normal; 1, lesion <10%; 2, 10–20% lesion; 3, 20–30% lesion; 4, 30–40% lesion; 5, >40% lesion (Yiu et al., 2016). For immunostaining of C3b and membrane attack complex (MAC), renal tissues were treated with 3% H_2_O_2_, blocked by 3% bovine serum albumin, and stained with anti-C3b antibody (sc-133172; Santa Cruz, CA, United States) and anti-MAC antibody (553,043; BD Pharmingen, NJ, United States) overnight at 4°C, respectively. Then the secondary antibody (PV-9002, PV-9004; ZSBIO, Beijing, China) was used, followed by color development using 3, 3′-diaminobenzidine (ZLI-9018; ZSBIO). The integrated optical density (IOD) was used to represent the deposition of C3 and MAC in kidneys. The images were analyzed by Image-Pro Plus under×200 magnification.

### Transmission Electron Microscopy

The renal cortical tissues of mice were cut into three 1 mm^3^ cubes and immediately immersed into 3% glutaraldehyde after sacrificed. Further sample handling was performed by the Laboratory of Electron Microscopy, Peking University First Hospital. Two glomeruli were randomly selected from each renal sample and a total of 15–20 representative non-overlapping digital micrographs from each glomerulus were taken on an electron microscope (Hitachi 7700 transmission, Tokyo) under×10,000 magnification. The number of foot process per micrometer of glomerular basement membrane (GBM) was calculated using Photoshop or ImageJ.

### Cell Culture

The primary renal proximal tubular cells were extracted from male *C57BL/6J* mice (3 weeks old) (Kamiyama et al., 2012). Cells were seeded in 6 or 12-well plates as needed and cultured in Epithelial Cell medium (EpiCM-A; ScienCell, United States) supplemented with 2% fetal bovine serum (FBS; ScienCell), 1% penicillin-streptomycin (ScienCell) and 1% EpiCGS-a (ScienCell) in 37°C in an incubator containing 5% CO_2_ and 95% humidified air. When reaching approximately 70% confluence, the medium was replaced, and the cells were exposed to different stimuli reagents for 24 h, including 30 mmol/L d-glucose (HG) (G8270, Sigma Aldrich), D-mannitol (MAN) (M4125, Sigma Aldrich) and dimethyloxallyl glycine (DMOG) (3695; Sigma-Aldrich, St Louis, United States).

### Western Blot Analysis

Sodium dodecylsulfate-polyacrylamide gel electrophoresis (SDS-PAGE) was used to separate denatured proteins. Total proteins were transferred electrophoretically to polyvinylidene difluoride transfer membranes (PVDF, Millipore, Bedford, MA, United States). The membrane was incubated with the primary antibody (HIF-1α 10006421; Cayman, United States, dilution 1:200), and then was incubated with a peroxidase-conjugated secondary antibody. Proteins were visualized on autoradiographic film using an ECL Plus Western blot detection system (GE Healthcare).

### Quantitative Real-Time Polymerase Chain Reaction Analysis

Total RNA of renal cortical tissues or cultured cells was extracted using RNAprep pure Kit (Tiangen, China) and reverse transcribed into cDNA with High-Capacity cDNA Reverse Transcription Kits (Applied Biosystems, MA, United States). The qRT-PCR analysis was carried out in an ABI Prism 7500 sequence detection system (Applied Biosystems) using SYBR green Master Mix (Applied Biosystems). Relative gene expression was normalized to 18s rRNA. Data were presented as a relative fold change compared with the control group. Primers used for mRNA detection are listed in Supplementary Table S1.

### Flow Cytometry Assay

Indirect labelling flow cytometry assay was used to measure Crry expression with a primary antibody (550058; BD Pharmingen), and then with a compatible secondary antibody (405407; APC Goat anti-rat IgG, Biolegend).

### Terminal-Deoxynucleotidyl Transferase Mediated Nick End Labeling Assay

Terminal-deoxynucleotidyl transferase mediated nick end labeling (TUNEL) assay was preformed using *In Situ* Cell Death Kit (Roche Diagnostics, Mannheim, Germany) to detect apoptotic cells according to the manufacturer’s instructions. Cells were incubated with 4% paraformaldehyde (PFA) for 20 min and permeabilized with 0.1% Triton X-100 for 15 min. Then, the specimens were incubated for 1 h in working strength TdT enzyme solution at 37°C. The slides were counterstained with 4′, 6-diamidino-2-phenylindole (DAPI).

### Statistical Analysis

The data were expressed as the means ± standard deviation. Parametric data were analyzed with one-way Anova (Tukey test). Non-parametric analyses of histological scores were conducted using a Spearman’s test with the unpaired, non-parametric Mann-Whitney *U* test as appropriate. *p* values < 0.05 were considered statistically significant. GraphPad prism 5.0 was used for statistical analysis.

## Results

### Dapagliflozin Ameliorated Diabetic Kidney Disease in db/db Mice

To confirm the effect of dapagliflozin *in vivo*, we treated type 2 diabetic *db/db* mice with dapagliflozin for 10 weeks. Compared with *m/m* mice, both vehicle-treated *db/db* mice and dapagliflozin-treated *db/db* mice had significantly higher body weight, kidney weight/body weight ratio, fasting blood glucose, urinary albumin creatinine ratio (UACR) and 24-h urinary albumin excretion. Dapagliflozin-treated *db/db* mice showed significantly lower levels of fasting blood glucose and urinary albumin excretion compared with vehicle-treated *db/db* mice (21.83 ± 6.05 vs. 15.46 ± 4.03 mmol/L, *p* = 0.044; 307.69 ± 98.34 vs. 174.72 ± 70.75 mg/24h, *p* = 0.011, respectively) (Table 1). Marginally significant decreasing of UACR was observed in dapagliflozin-treated *db/db* mice compared with vehicle-treated *db/db* mice (385.53 ± 178.27 mg/g Cr vs. 230.40 ± 72.48 mg/g Cr, *p* = 0.054). No adverse event was found in any experimental group.

**TABLE 1:** Laboratory data of mice.

	*m/m* mice	Vehicle treated *db/db* mice	SGLT2i treated *db/db* mice
body weight, g	25.4 ± 1.77	49.96 ± 4.56**	55.38 ± 5.76
kidney weight/body weight	0.0058	0.0044*	0.0040
water intake, ml/24h	5.60 ± 1.08	24.35 ± 8.74**	20.43 ± 9.75
food intake, g/24h	4.48 ± 0.48	14.16 ± 1.42**	14.11 ± 1.88
urine volume, ml/24h	0.83 ± 0.18	17.07 ± 7.01**	16.29 ± 5.24
fasting blood glucose, mmol/L	7.90 ± 1.28	21.83 ± 6.05**	15.46 ± 4.03^†^
UACR, ug/mg	75.34 ± 22.74	385.53 ± 178.27**	230.40 ± 72.48
urine total albumin, mg/24h	20.96 ± 5.07	307.69 ± 98.34**	174.72 ± 70.75^†^

Metabolic parameters and urine test of *m/m*, *db/db* mice and Dapagliflozin-treated *db/db* mice. Four-week-old *db/db* mice (*n* = 7) were treated with 1 mg/kg/day Dapagliflozin for 10 weeks. Values are means ± SD. UACR: urinary albumin creatinine ratio; *m/m* mice:C57BL/KsJ mice.

*p < 0.05, **p < 0.01 compared with *m/m* mice (*n* = 5).

† *p* < 0.05 compared with vehicle-treated *db/db* mice (*n* = 7).

**FIGURE 1:**
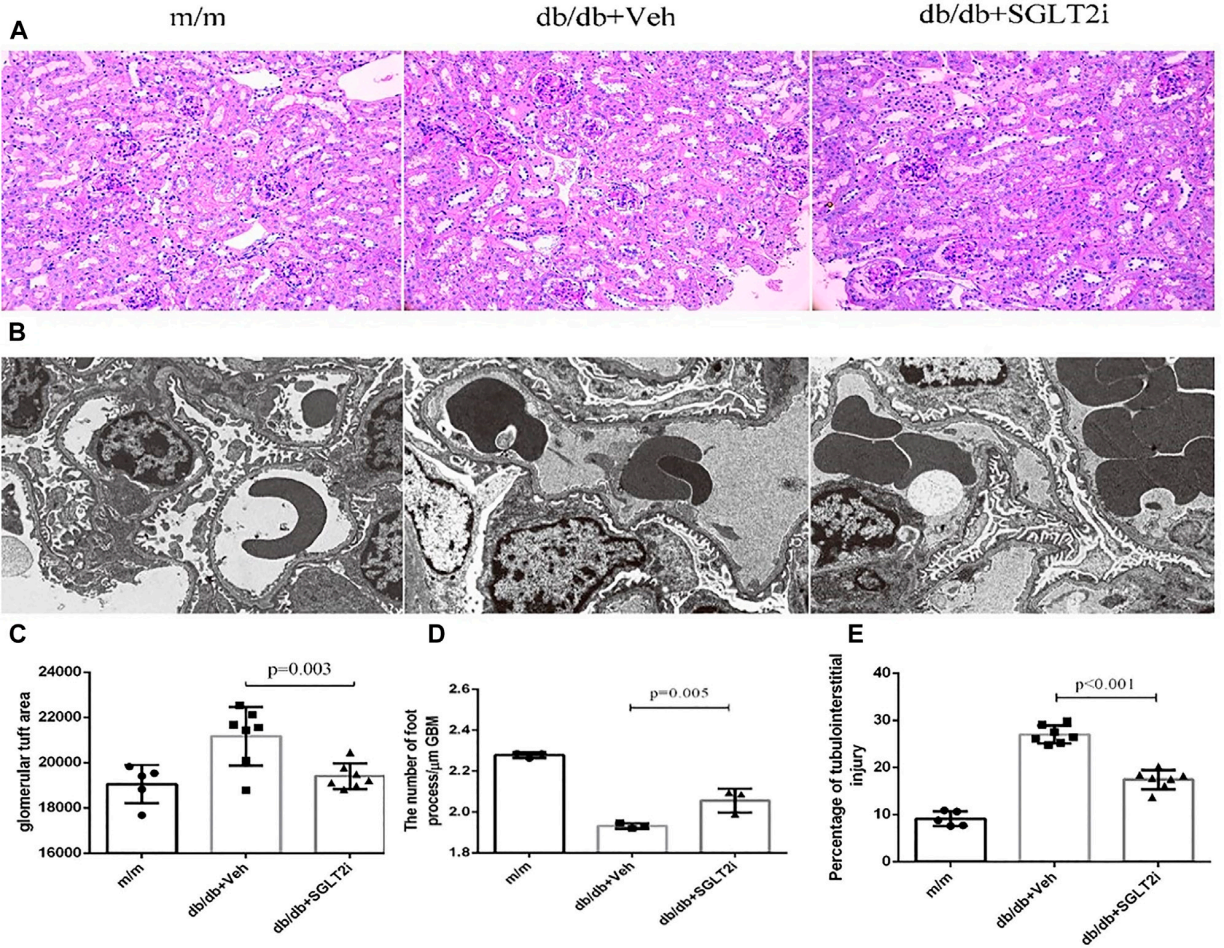
Dapagliflozin ameliorated diabetic kidney disease in *db/db* mice. **(A)** Representative PAS staining of kidney biopsy from *m/m* mice (*n* = 5), vehicle-treated *db/db* mice (*n* = 7) and SGLT2i-treated *db/db* mice (*n* = 7) showing mesangial matrix expansion, Bar = 100 μm. **(B)** Representative ultrastructure changes of GBM by electron microscopy under ×10000 magnification, *n* = 3 for each group. **(C)** Semiquantitative analysis of mesangial matrix expansion. **(D)** The number of foot processes. **(E)** Semiquantitative analysis of tubulointerstitial injury. PAS, periodic acid-Schiff; SGLT2i, sodium-glucose cotransporter 2 inhibitor; GBM, glomerular basement membrane; MPTEC, mouse proximal tubular epithelial cell; DMOG, dimethyloxallyl glycine.

In PAS staining, notably increased mesangial matrix expansion and tubulointerstitial injury were detected in *db/db* mice compared with *m/m* controls (*p* < 0.001 and *p* < 0.001, respectively, Figures 1A,C,E). The ratio of mesangial matrix expansion area to the glomerular tuft area and tubulointerstitial injury were significantly attenuated in dapagliflozin-treated *db/db* mice compared with vehicle-treated *db/db* mice (*p* = 0.003, *p* < 0.001, respectively, Figures 1A,C,E).

By electron microscopy, dapagliflozin-treated *db/db* mice showed significant improvement in thickening of GBM compared with vehicle-treated db/db mice (*p* = 0.005, Figures 1B,D).

Collectively, dapagliflozin ameliorated diabetic kidney disease in *db/db* mice including laboratory and pathological indexes.

### Dapagliflozin Upregulated Crry Expression and Suppressed Kidney Complement Over-Activating in db/db Mice

**FIGURE 2:**
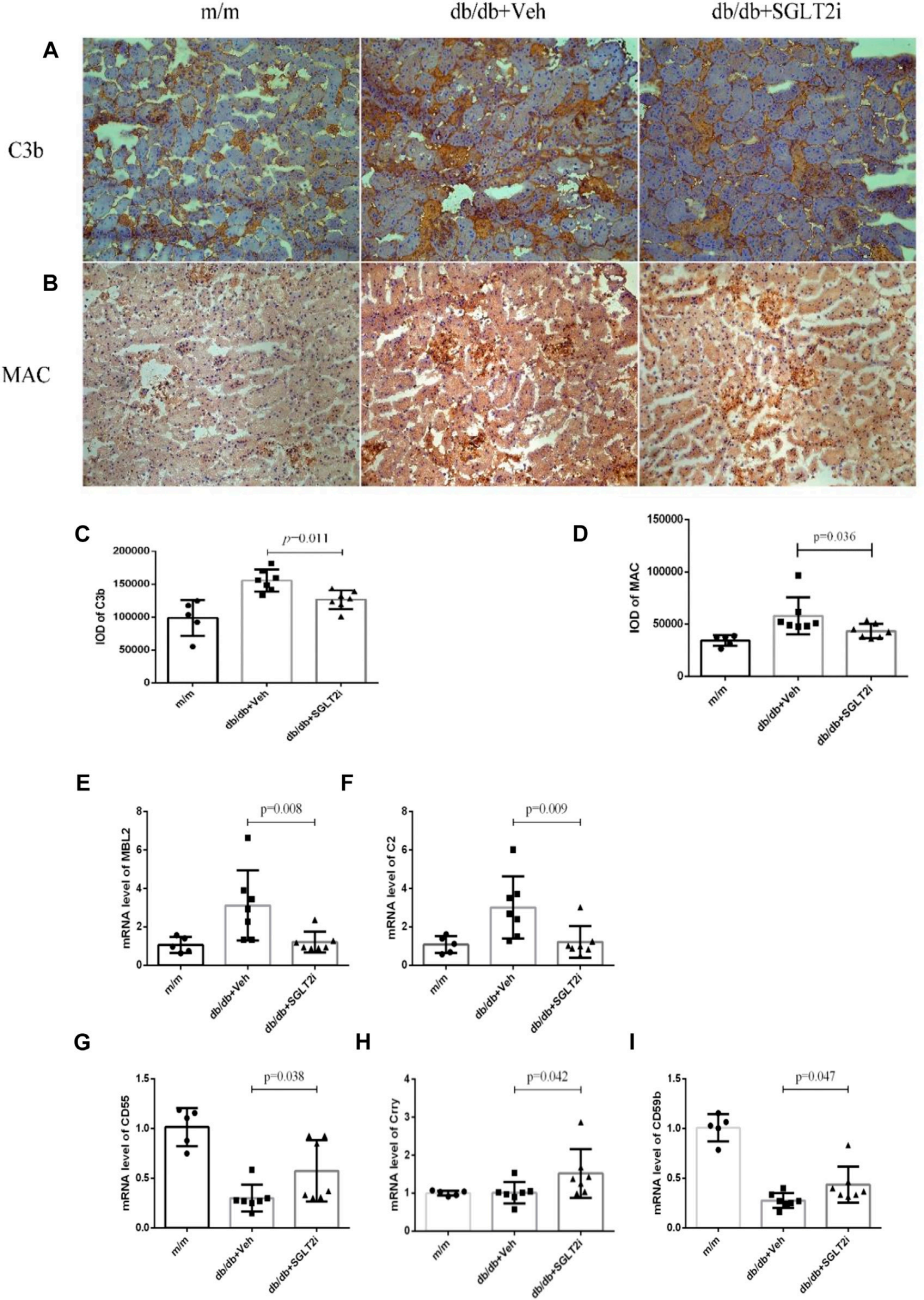
Dapagliflozin suppressed kidney complement over-activating in *db/db* mice and upregulated Crry expression. **(A)** Representative immunohistochemical staining of C3b from *m/m* mice (*n* = 5), vehicle-treated *db/db* mice (*n* = 7) and SGLT2i-treated *db/db* mice (*n* = 7), Bar = 100 μm. **(B)** Representative immunohistochemical staining of MAC. **(C)** Integral optical density of C3b deposition. **(D)** Integral optical density of MAC deposition. Real-time qPCR analysis of mRNA level change **(E)** MBL, **(F)** C2, **(G)** CD55, **(H)** Crry, **(I)** CD59. MBL, mannan binding lectin; C2, complement 2; MAC, membrane attack complex.

Then we investigated the effect of dapagliflozin on the complement system in diabetic mice. Both C3b and MAC deposition were significantly reduced in dapagliflozin-treated *db/db* mice compared with vehicle-treated *db/db* mice, as demonstrated by immunohistochemistry analysis (*p* = 0.011 and *p* = 0.036, respectively) (Figures 2A–D). The mRNA levels of mannan binding lectin (MBL) and C2 were also significantly reduced in dapagliflozin-treated *db/db* mice compared with vehicle-treated *db/db* mice (*p* = 0.008 and *p* = 0.009, respectively, Figures 2E,F).

Compared with vehicle-treated *db/db* mice, the mRNA levels of complement regulatory proteins including CD55, Crry and CD59 were significantly upregulated in dapagliflozin-treated *db/db* mice (*p* = 0.038, *p* = 0.042 and *p* = 0.047, respectively, Figures 2G–I).

### Dapagliflozin Relieved Tubular Cell Injury by Upregulating Crry and Inhibiting High Glucose-Induced HIF-1α Protein Accumulation *in vitro*

**FIGURE 3:**
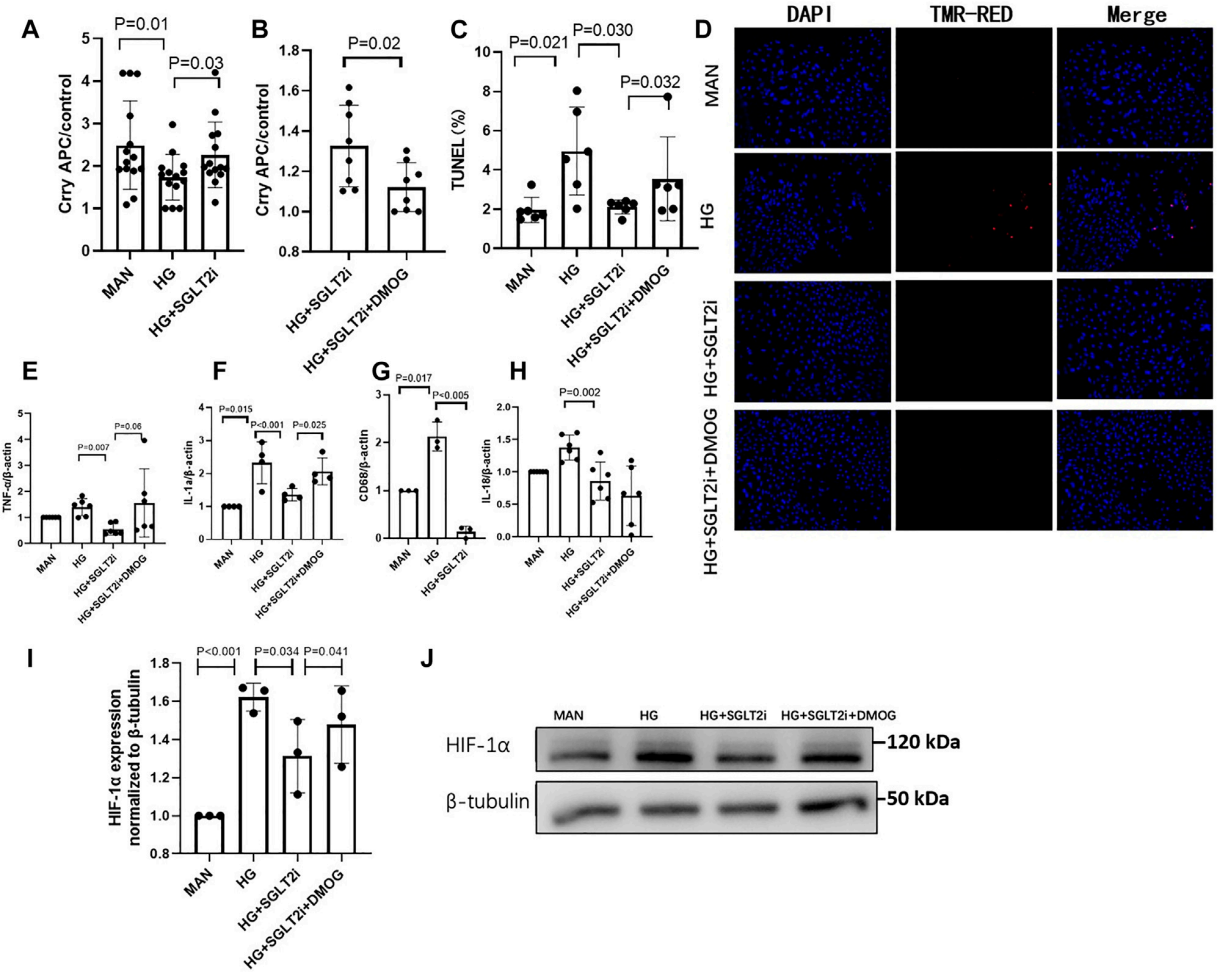
Dapagliflozin relieved tubular injury by upregulating Crry and inhibiting high glucose-induced HIF-1α accumulation *in vitro*. **(A)** In MPTECs, dapagliflozin upregulated Crry under HG condition (30 mmol/L). **(B)** The upregulation of Crry was significantly reduced by DMOG (1 mmol/L), a HIF-1α stabilizer. **(C,D)** The apoptosis ratio was analyzed by TUNEL assay. Dapagliflozin significantly reduced apoptosis ratio of MPTECs and the protective effect was reduced by DMOG. Real-time qPCR analysis of mRNA level change **(E)** TNF-α, **(F)** IL-1α, **(G)** CD68, **(H)** IL-18, **(I)** and **(J)** Protein expression levels of HIF-1α were determined by western blot analysis and quantitated by densitometry. MPTECs, mouse primary proximal tubular epithelial cells; DMOG, dimethyloxallyl glycine.

As mentioned above, Crry is a key complement regulator in rodents, with similar function to MCP and CD55 in human (Li et al., 2019b; Qin et al., 2004; Ostergaard et al., 2007). In order to further demonstrate whether dapagliflozin can upregulate Crry protein expression, we utilized the mouse primary tubular epithelial cells (MPTECs). Under high glucose condition (30 mmol/L), the expression of Crry in MPTECs was significantly reduced (*p* = 0.012, compared with D-mannitol-treated MPTECs, Figure 3A). The reduced Crry was upregulated by dapagliflozin (dapagliflozin 100 μmol/L, this dose was selected according to previous studies) (Bessho et al., 2019; Cai et al., 2020; Li et al., 2020) under HG condition (*p* = 0.031, compared with vehicle-treated MPTECs, Figure 3A). In line with the increasing of Crry, key markers of inflammation including TNF-α, IL-1α, CD68 and IL-18 under HG condition were reduced in mRNA level in MPTECs treated with dapagliflozin, as compared with vehicle-treated MPTECs (Figures 3E–H). Moreover, under high glucose condition, the TUNEL assay revealed significantly lower level of apoptosis in dapagliflozin-treated MPTECs compared with the vehicle-treated MPTECs (*p* = 0.034, Figures 3C,D).

Furthermore, we investigated whether HIF-1α accumulation under high glucose condition was relieved by dapagliflozin, and whether dapagliflozin upregulated Crry expression *via* decreasing HIF-1α. HIF-1α accumulation was stabilized in MPTECs by DMOG (1 mmol/L), a prolyl hydroxylase (PHD) inhibitor. When MPTECs were treated with DMOG based on high glucose and dapagliflozin, Crry expression was remarkably reduced with the stabilization of HIF-1α accumulation (*p* = 0.025 and *p* = 0.041, respectively, compared with high glucose and dapagliflozin-treated MPTECs without DMOG, Figures 3B,I,J), indicating the effect of dapagliflozin on upregulating Crry expression was reduced by HIF-1α stabilizer. With the reducing of Crry, the above markers of inflammation and apoptosis significantly increased (Figures 3E,F,H).

Collectively, dapagliflozin restored Crry protein expression *via* reducing HIF-1α accumulation in MPTECs.

## Discussion

The current study found that the SGLT2 inhibitor alleviates complement over-activation by upregulating Crry expression *via* reducing HIF-1α accumulation in renal proximal tubular cells in DKD.

Increasing evidence demonstrated that the inflammatory process mediated mainly by innate immunity is among the central pathophysiology of DKD (Tang and Yiu, 2020). As a key part of innate immunity, the complement system is actively involved in the pathogenesis of DKD (Tang and Yiu, 2020; Kolev et al., 2014; Nangaku, 1998). Our previous studies also showed that complements were over-activated in DKD (Li et al., 2019a; Li et al., 2019b; Sun et al., 2019). Inhibiting complement over-activation is a potential therapeutic strategy for DKD.

In the current study, we found that dapagliflozin treatment significantly reduced C3b and MAC deposition in kidney tissues of *db/db* mice. Further investigation suggested that such alleviation of complement over-activation by dapagliflozin was associated with the upregulating of Crry. The murine cell surface protein Crry is a key complement regulator with similar function to MCP and CD55 in human. Previous studies found that Crry is critical for regulating complement activation within kidney tubules (Nangaku, 2006; Thurman et al., 2006). During ischemia-reperfusion challenge, Crry also has a critical role in protecting proximal tubular epithelial cells (Thurman et al., 2006). We speculated that in human DKD, dapagliflozin might improve DKD by, at least in part, upregulating MCP or CD55, and thus relieves complement over-activation.

Tubular injury is an important aspect of DKD, and tubular hypoxia is the main driving force for proximal tubulopathy (Mimura and Nangaku, 2010). Hyperglycemia induces glomerular hyperfiltration and increases tubular reabsorption of sodium and glucose through SGLTs, which enhance the activity of sodium-potassium-ATPase, resulting in increased oxygen and energy consumption. Thus, proximal tubular cells in diabetic kidneys are exposed to chronic hypoxia. For rodent models, previous studies demonstrated that SGLT2 inhibitors ameliorated hypoxia in the kidney cortex (Layton et al., 2016; O'Neill et al., 2015). HIF-1α was proposed as a therapeutic target of the SGLT2 inhibitor for DKD (Bessho et al., 2019; Cai et al., 2020; Li et al., 2020). In this study, we found that dapagliflozin decreased HIF-1α expression, leading to upregulation of Crry. HIF-1α is an important transcription factor of mammalian oxygen sensing. Under hypoxia condition, HIF-1α can maintain stable and bind with HIF-1β, then activate and increase gene transcription (O'Neill et al., 2015; Majmundar et al., 2010; Kaelin and Ratcliffe, 2008). It can regulate glucose metabolism, cell proliferation, angiogenesis proteins and inflammation (Dengler et al., 2014). As a key part of innate immunity, complement over-activation plays an important role in the pathogenesis of DKD. A previous study showed an important role for HIF-1α in regulating CD55 expression on airway epithelium *in vitro* and *in vivo* (Pandya et al., 2016), which prompts us to investigate whether dapagliflozin upregulates Crry *via* relieving HIF-1α accumulation in the kidney.

To our knowledge, this is the first study investigating the relationship between HIF-1α and complement regulators in kidneys. The exact molecular mechanism of HIF-1α regulating complement regulator expression is not fully clear. One speculation is that transcriptional downregulation may occur through epigenetic modifications. Hypoxia targets Jumonji AT-rich interactive domain 1A (JARID1A) activity, which in turn increases Histone H3 lysine 4 (H3K4) trimethylation (H3K4me3), leading to the altered programs of gene expression including CD55 (Zhou et al., 2010; Kiernan et al., 2020). The current study found that dapagliflozin reduced HIF-1α accumulation, upregulated Crry and ameliorated complement over-activation; with the accumulation of HIF-1α stabilized by DMOG, the expression of Crry was decreased. The upregulation of Crry expression by dapagliflozin might improve DKD, at least in part, *via* strengthening the key protective defense on complement over-activation which was added to the hypoglycemic effect. It should be note that no obvious change for Crry mRNA between *m/m* and *db/db* mice was observed in the current study, indicating that there is little, if any, pathological relevance associated with Crry in kidneys of *db/db* mice. Therefore, the effect of dapagliflozin on upregulating Crry expression might be achieved through the pharmacological way rather than the physiological way.

There are several limitations of our study. First, isoforms of HIFs other than HIF-1α exist, such as HIF-2α and HIF-3α. We focused on HIF-1α in our studies due to its ubiquitous expression and its strong induction during hypoxia. However, the current study does not exclude the possibility of other HIF isoforms in regulating Crry. Second, the effect of dapagliflozin on other intrinsic cells of kidneys including mesangial cells and podocytes was not clarified. Third, the numbers of animals included is limited, studies with larger sample size are needed.

In conclusion, this study provides a novel mechanism of the protective role of SGLT2 inhibitors in DKD. Clarifying the molecular regulatory mechanisms of complement regulators and HIF-1α expression by SGLT2 inhibitors will lead to improvement to manage DKD(Singh et al., 2020).

## Data Availability

The raw data supporting the conclusion of this article will be made available by the authors, without undue reservation.
