# Supplementary material for: Dapagliflozin Ameliorates Diabetic Kidney Disease via Upregulating Crry and Alleviating Complement Over-activation in db/db Mice
Source: Front Pharmacol. 2021 Oct 12;12:729334. doi: 10.3389/fphar.2021.729334 (PMC8546210; doi:10.3389/fphar.2021.729334)
Supplement: Supplementary file 1 [file Table1.DOCX]

| 18S F | GTAACCCGTTGAACCCATT |
| --- | --- |
| 18S R | CCATCCAATCGGTAGTAGCG |
| TNFα F | TGTCTACTCCCAGGTTCTCT |
| TNFα R | GGGGCAGGGGCTCTTGAC |
| CD68 F | CATCAGAGCCCGAGTACAGTCTACC |
| CD68 R | AATTCTGCGCCATGAATGTCC |
| IL18 F | GACTCTTGCGTCAACTTCAAGG |
| IL18 R | CAGGCTGTCTTTTGTCAACGA |
| IL1α F | CGAAGACTACAGTTCTGCCATT |
| IL1α R | GACGTTTCAGAGGTTCTCAGAG |
| Crry F | AGGTCTCTTCTCGGAGTTCAG |
| Crry R | AGGTCTCTTCTCGGAGTTCAG |
| C2 F | CGGTGGTAATTTCACCCTCAG |
| C2 R | GGTGTGATGTGAGCTAGACCT |

Supplement Table 1. Primers used for mRNA detection
