# Supplementary figures and images for: Dapagliflozin Ameliorates Diabetic Kidney Disease via Upregulating Crry and Alleviating Complement Over-activation in db/db Mice
Source: Front Pharmacol. 2021 Oct 12;12:729334. doi: 10.3389/fphar.2021.729334 (PMC8546210; doi:10.3389/fphar.2021.729334)

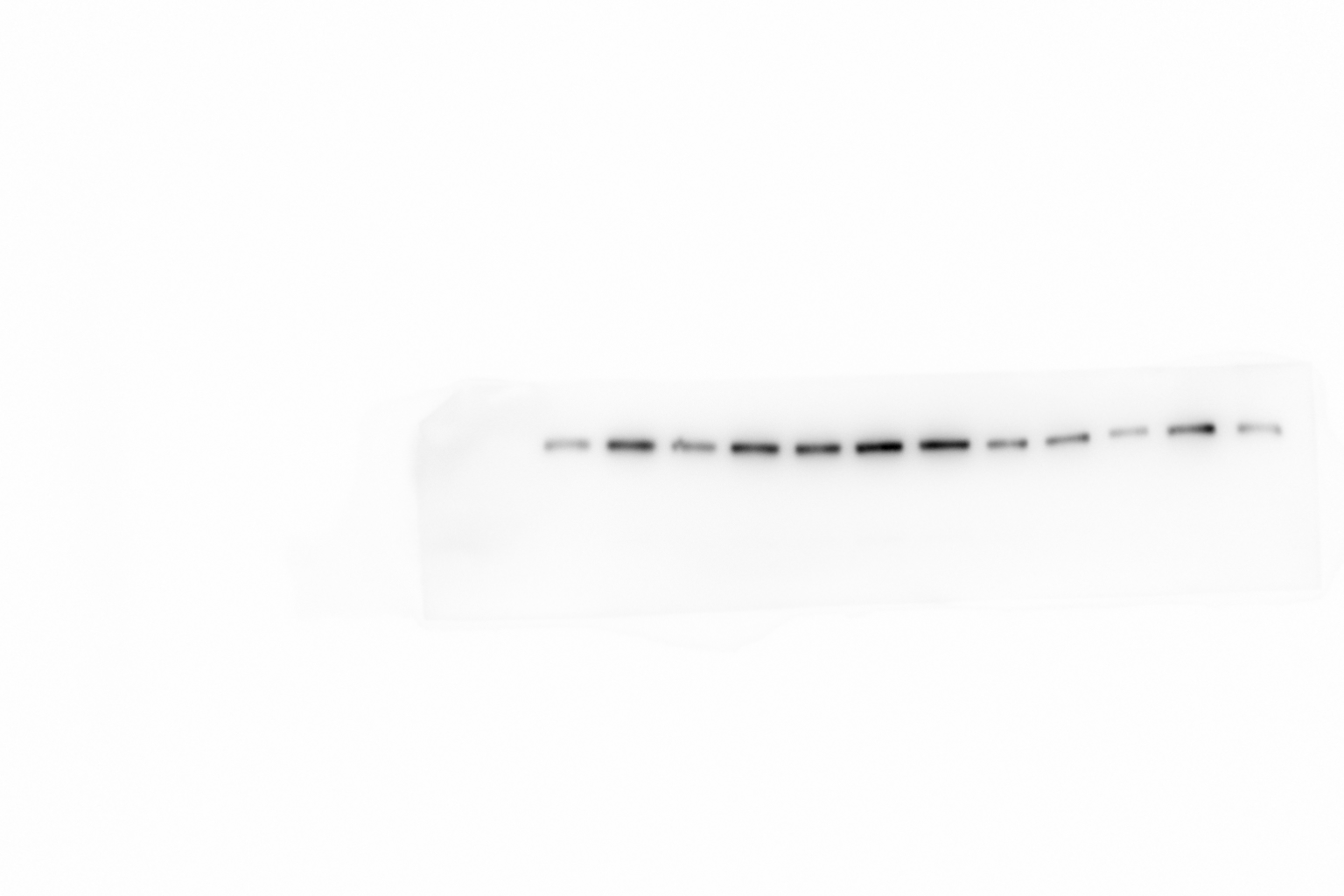

Supplement: Supplementary file 2 [file DataSheet1.ZIP › raw data/in vitro/WB/20201231_1204 tubulin.gel]

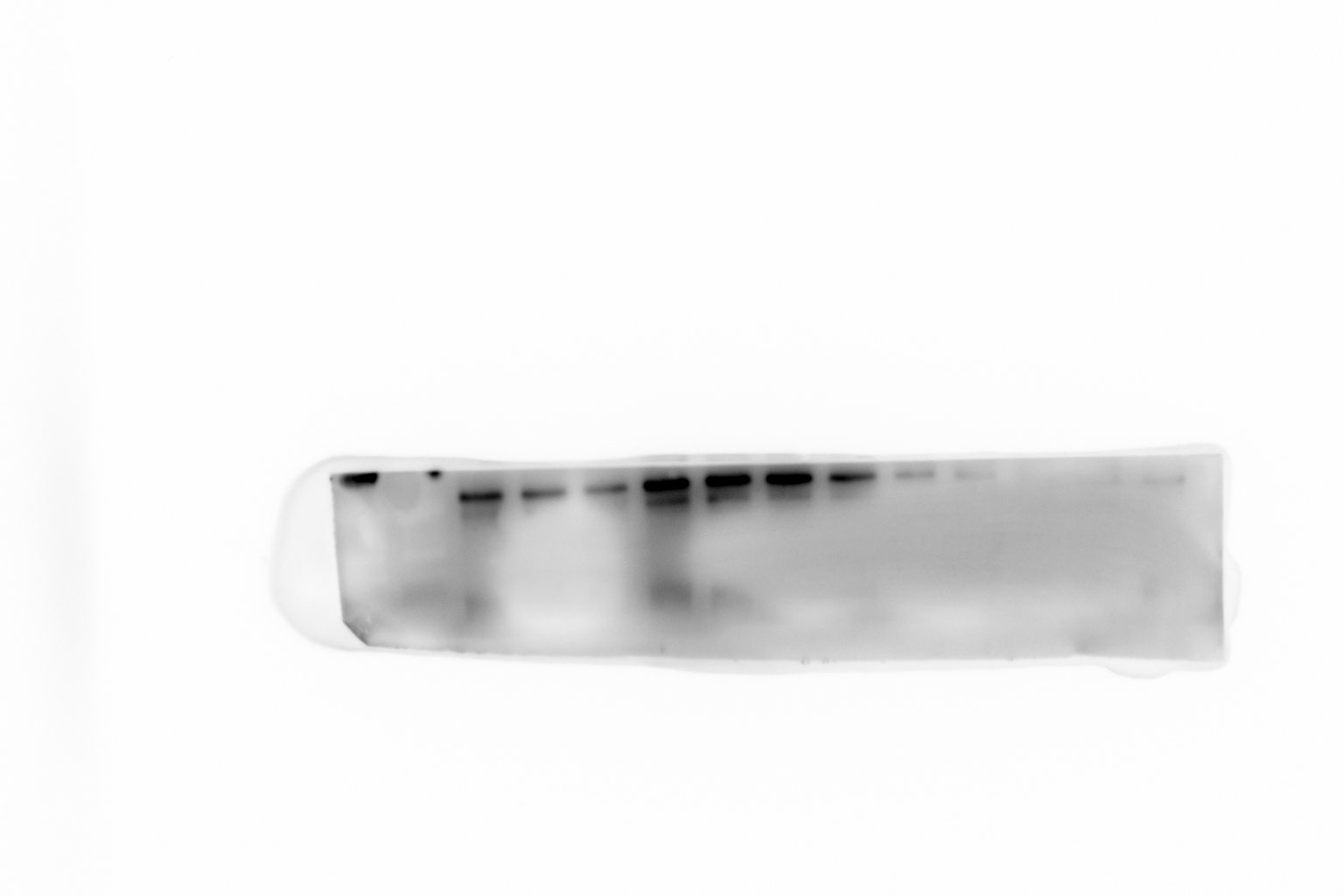

Supplement: Supplementary file 2 [file DataSheet1.ZIP › raw data/in vitro/WB/20201231_1204 HIF1a.gel]

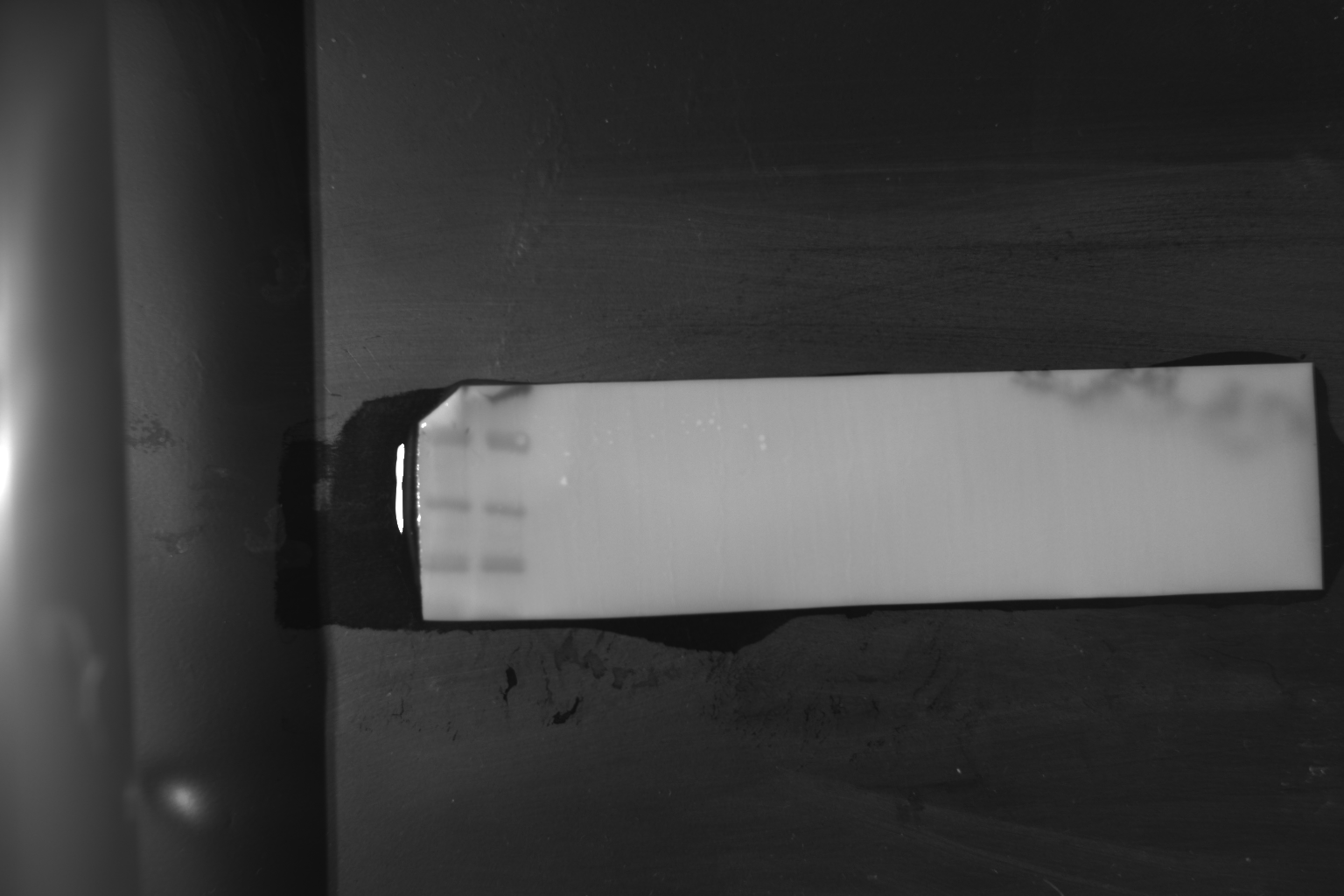

Supplement: Supplementary file 2 [file DataSheet1.ZIP › raw data/in vitro/WB/20201231_1204protein tubulin.gel]

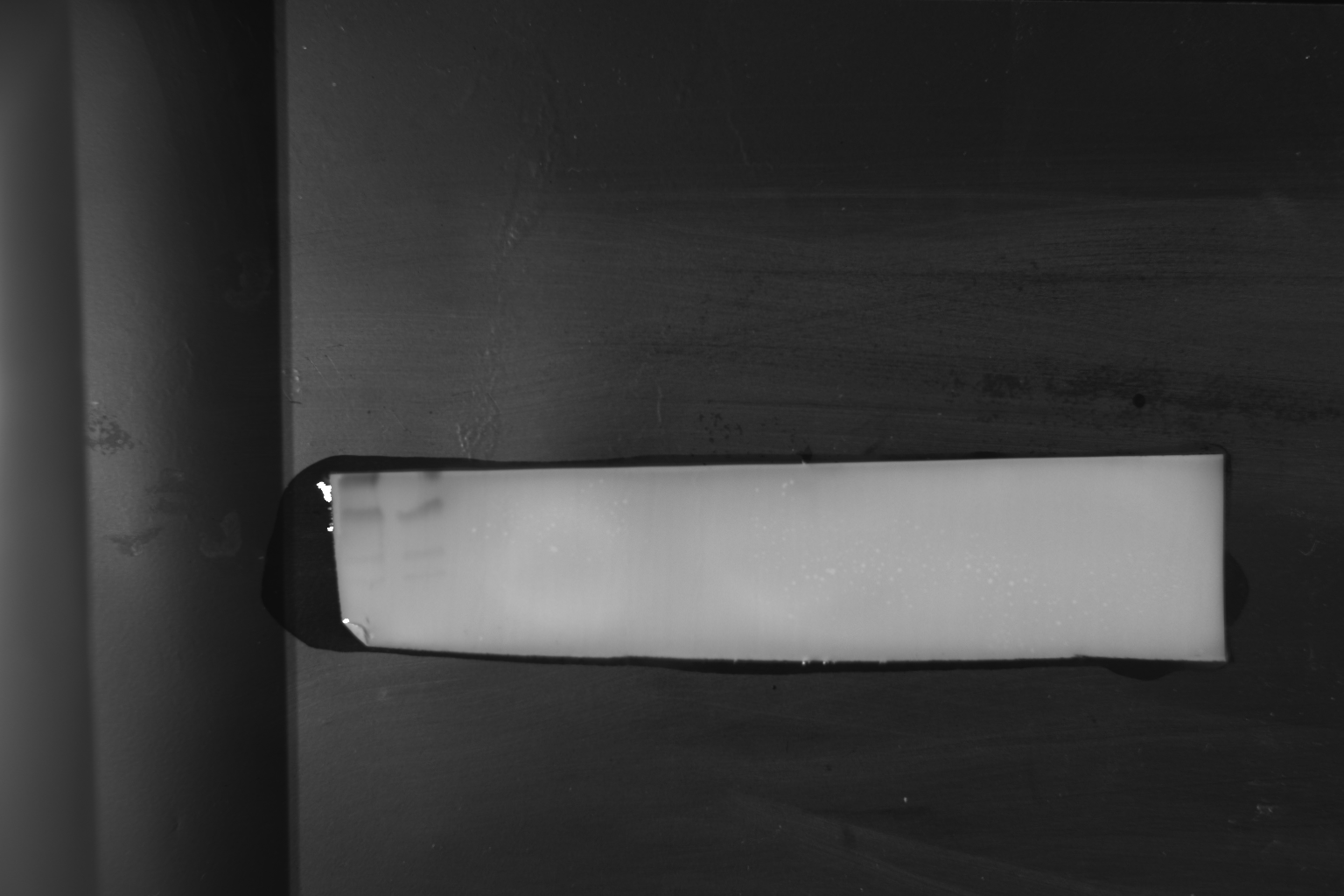

Supplement: Supplementary file 2 [file DataSheet1.ZIP › raw data/in vitro/WB/20201231_1204protein.gel]

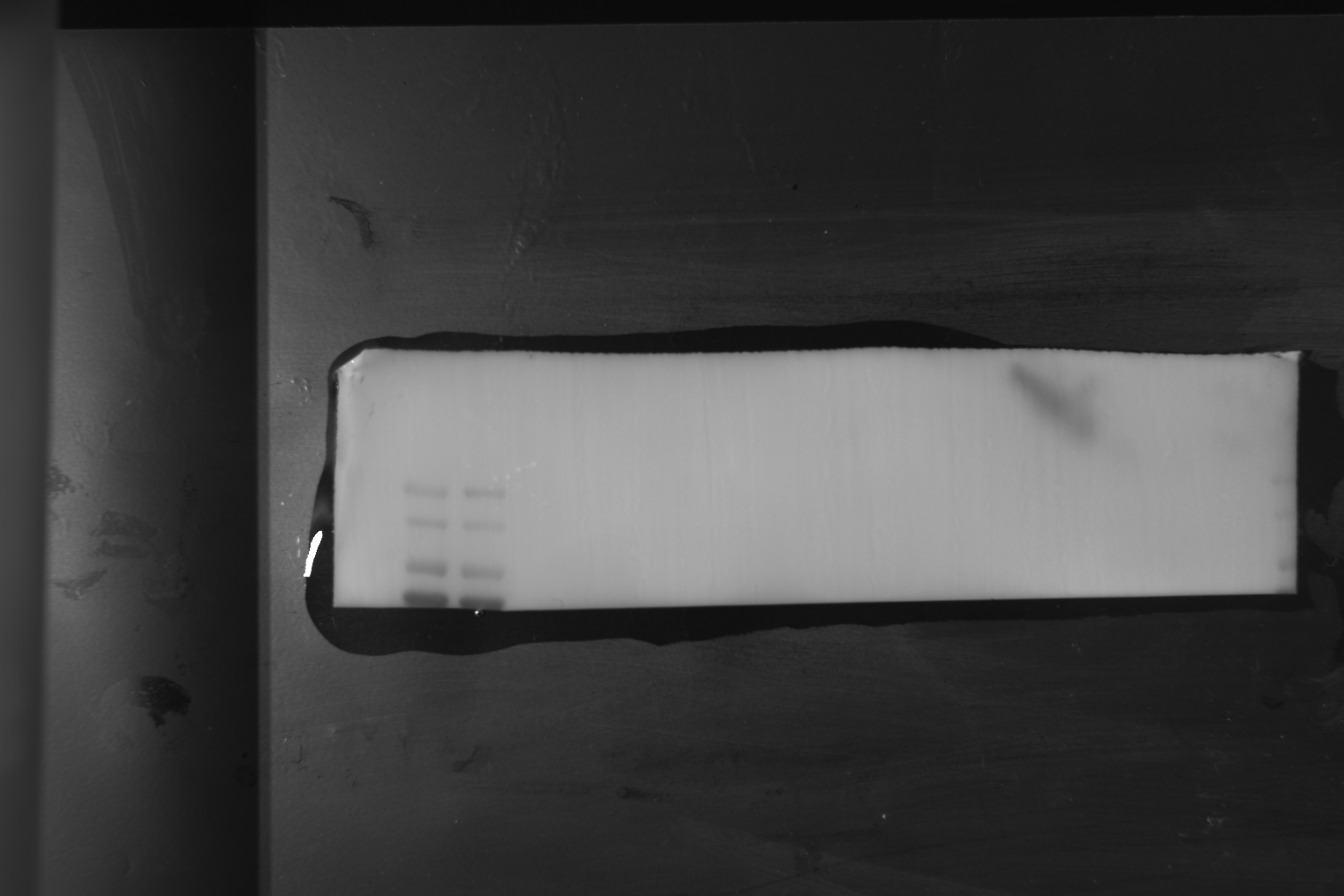

Supplement: Supplementary file 2 [file DataSheet1.ZIP › raw data/in vitro/WB/20201231_1230 protein h marker.gel]

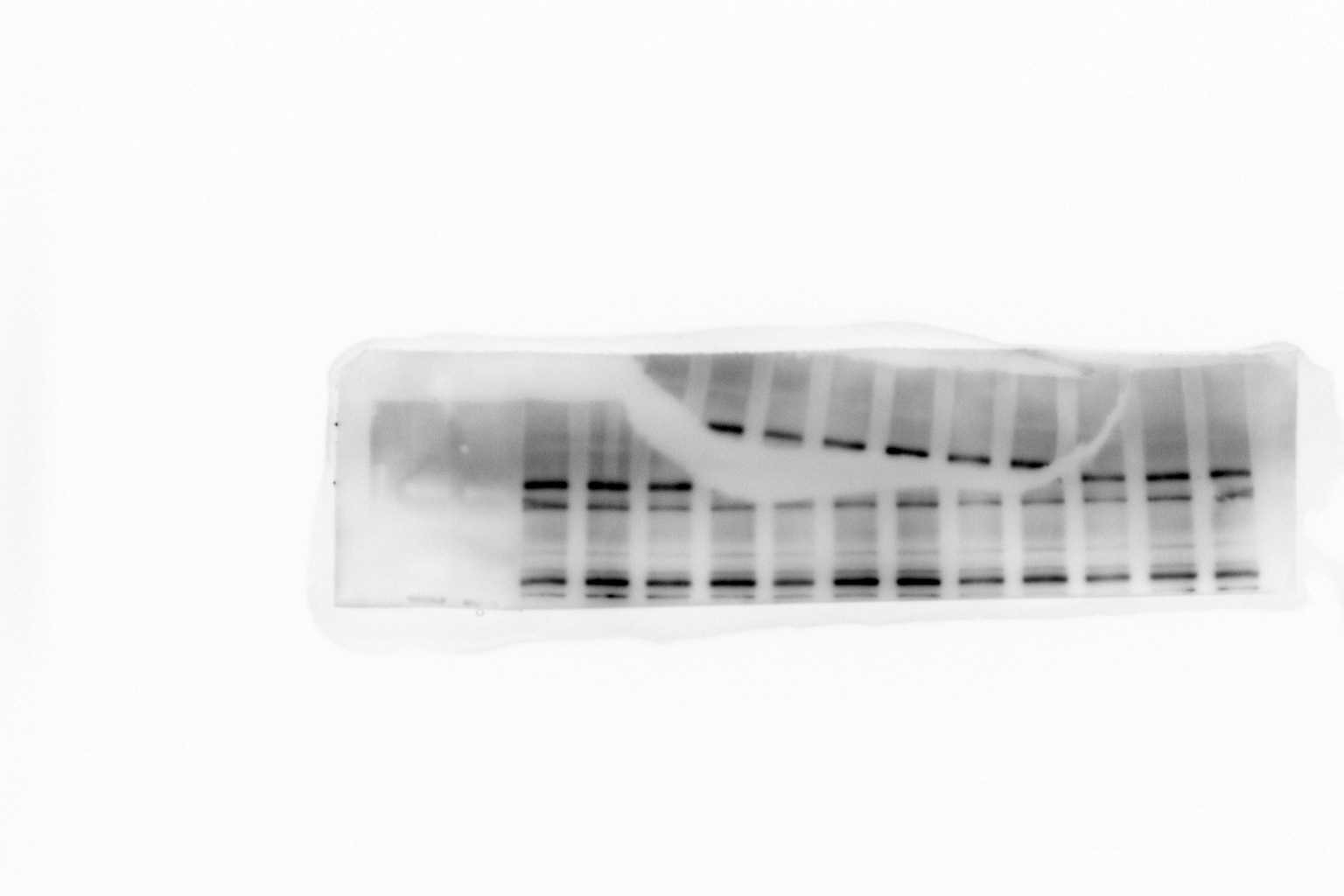

Supplement: Supplementary file 2 [file DataSheet1.ZIP › raw data/in vitro/WB/20201231_1230 protein HIF1a.gel]

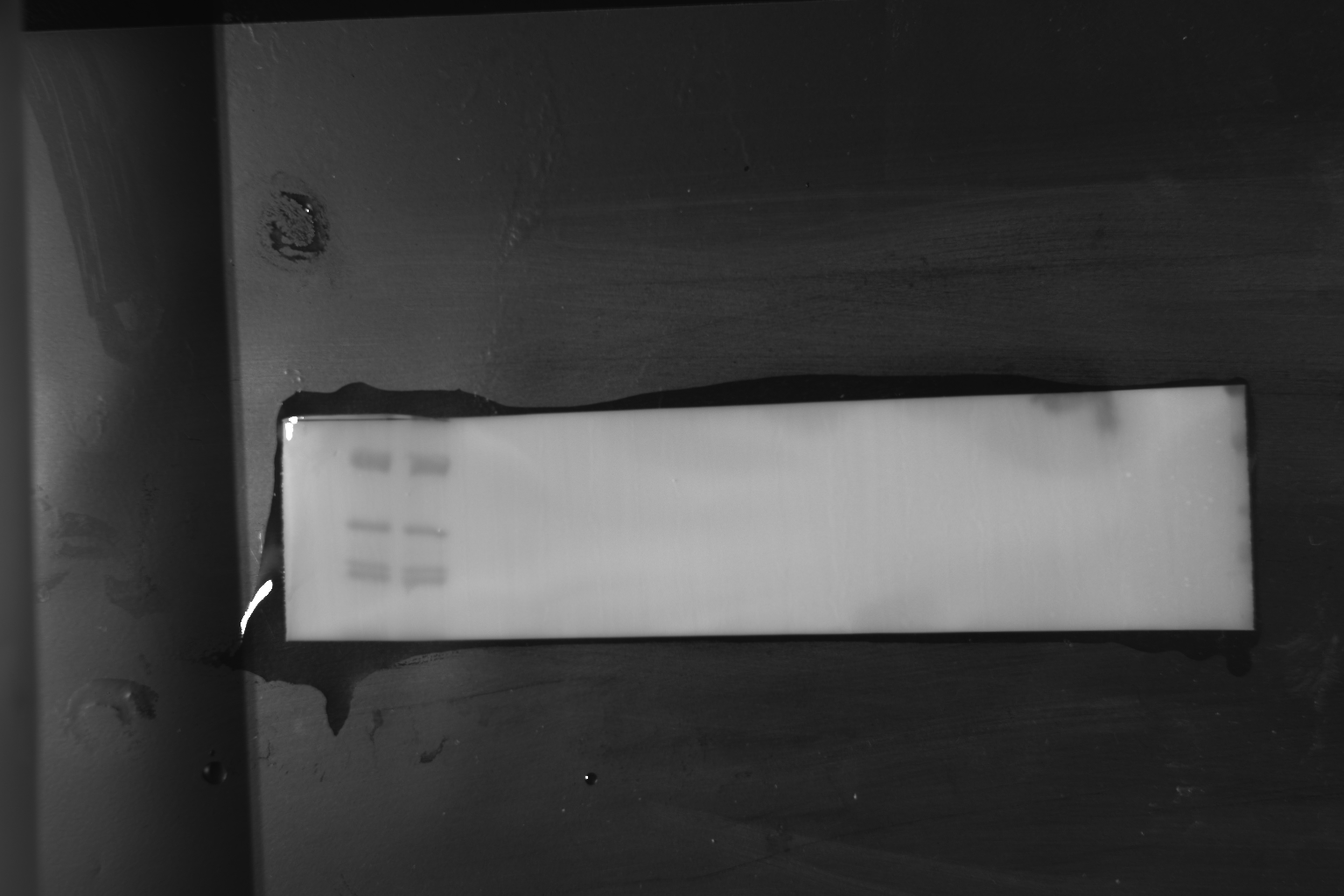

Supplement: Supplementary file 2 [file DataSheet1.ZIP › raw data/in vitro/WB/20201231_1230 protein t marker.gel]

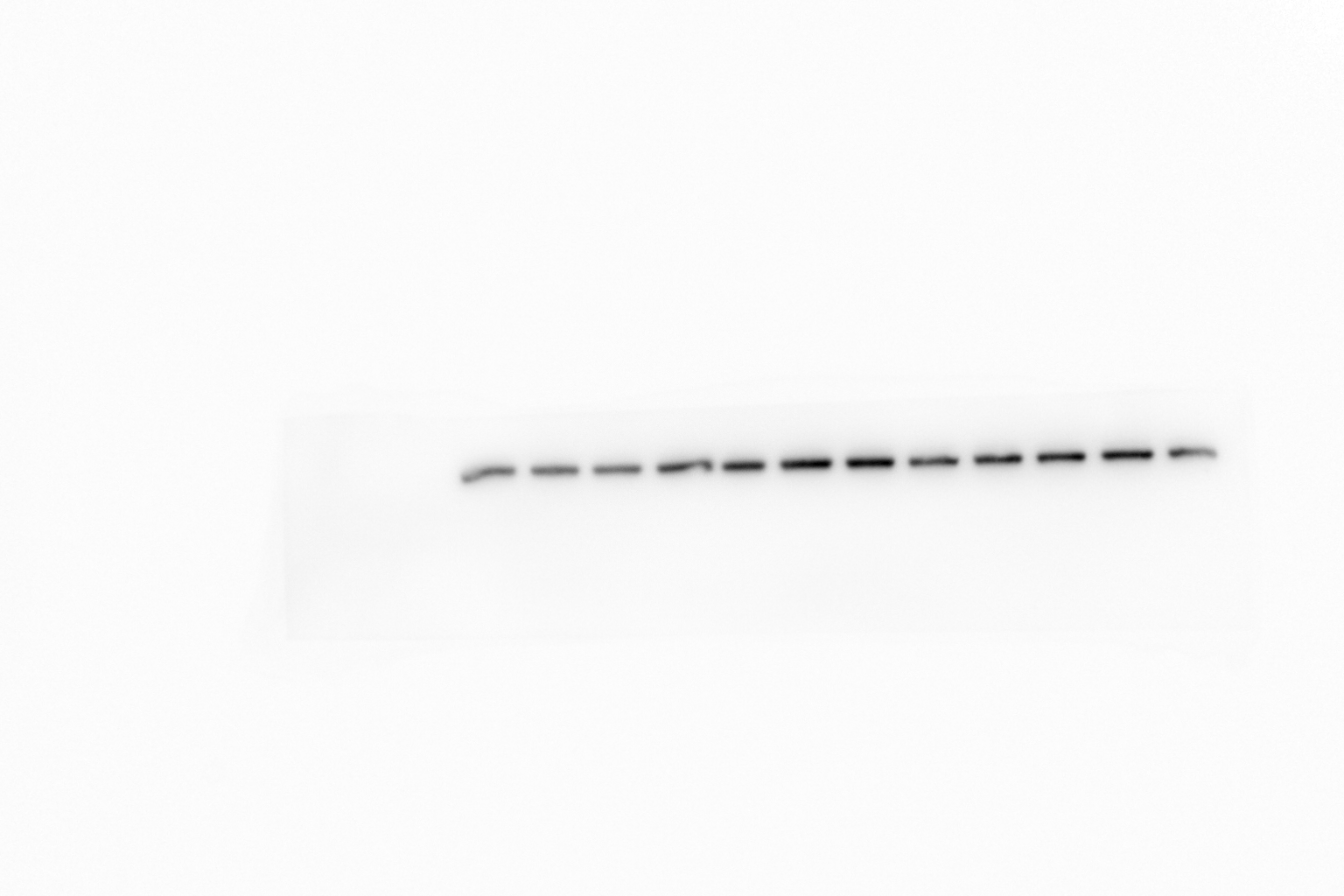

Supplement: Supplementary file 2 [file DataSheet1.ZIP › raw data/in vitro/WB/20201231_1230 protein tubulin.gel]
